# Transcriptome Comparison between Porcine Subcutaneous and Intramuscular Stromal Vascular Cells during Adipogenic Differentiation

**DOI:** 10.1371/journal.pone.0077094

**Published:** 2013-10-10

**Authors:** Shuzhong Jiang, Hongkui Wei, Tongxing Song, Yang Yang, Jian Peng, Siwen Jiang

**Affiliations:** 1 Department of Animal Nutrition and Feed Science, College of Animal Science and Technology, Huazhong Agriculture University, Wuhan, People’s Republic of China; 2 Key Laboratory of Swine Genetics and Breeding of Agricultural Ministy, and Key Laboratory of Agricultural Animal Genetics, Breeding and Reproduction of Ministry of Education, College of Animal Science and Technology, Huazhong Agriculture University, Wuhan, People’s Republic of China; Harbin Institute of Technology, China

## Abstract

Intramuscular fat (IMF) is an important trait influencing meat quality, and preadipocyte differentiation is a key factor affecting IMF deposition. Here we compared the transcriptome profiles of porcine intramuscular and subcutaneous preadipocytes during differentiation to gain insight into specific molecular and cellular events associated with intramuscular stromal vascular cell (MSVC) differentiation. RNA-Seq was used to screen for differentially expressed genes (DEGs) during the *in vitro* differentiation of MSVC and subcutaneous stromal vascular cell (ASVC) on days 0, 2 and 4. A total of 985 DEGs were identified during ASVC differentiation and 1469 DEGs during MSVC differentiation. Among these DEGs, 409 genes were specifically expressed during ASVC differentiation, 893 genes were specifically expressed during MSVC differentiation, and 576 DEGs were co-expressed during ASVC and MSVC differentiation. The expression profiles of DEGs during ASVC or MSVC differentiation were determined by cluster analysis based on Short Time-series Expression Miner (STEM). Four significant STEM profiles (profiles 1, 4, 5, and 14) were determined during ASVC differentiation, and four significant STEM profiles (profiles 1, 4, 11, and 14) were determined during MSVC differentiation. Gene ontology (GO) analysis indicated that DEGs related to adipocyte differentiation were identified to be significantly enriched in both adipose and muscle profile 14. In addition, Kyoto Encyclopedia of Genes and Genomes (KEGG) pathway analysis of DEGs in adipose profile 14 and muscle profiles 11 and 14 (STEM clustered them into one cluster) showed that the PPAR signaling pathway was significantly enriched in these profiles and four signaling pathways were specifically enriched in muscle profiles 11 and 14. Furthermore, analysis of transcription factor binding sites (TFBS) in the gene set revealed two over-represented transcription factors (NR3C4 and NR3C1), which were specifically significantly enriched in the promoter regions of genes within muscle gene expression profiles 11 and 14.

## Introduction

The deposition of intramuscular fat in pigs significantly contributes to meat quality, including juiciness, flavor, and tenderness[1]. To meet the increasing demand of consumers for high-quality pork, a main goal of animal scientists worldwide is to improve IMF content. The increase of adipose tissue mass is a result of adipocyte hyperplasia (increase in number) and hypertrophy (increase in size) [2], but the ratio between the two processes varies among depots [3]. Previous study has suggested that IMF deposition appeared to mainly depend on hyperplasia [4]. Thus, to increase the IMF content, the potential factors influencing intramuscular adipocyte hyperplasia that occurs via the differentiation of preadipocytes into adipocytes should be investigated. Interestingly, preadipocytes in different fat depots appear to have distinct adipogenic potential [5]. However, the tissue-specific molecular regulatory mechanisms underlying intramuscular preadipocyte differentiation remain unclear.

Primary preadipocyte differentiation is the transformation of a fibroblast-like cell to a lipid-filled cell [2]. The process is complex and can be initiated by exposure to many adipogenic stimuli such as glucocorticoids, IGF-1, and other hormones [6]. These stimuli activate signaling pathways that regulate the expression and activity of a set of differentiation-related transcription factors that then lead to the expression of downstream differentiation-specific genes [7]. Identifying the differentially expressed genes (DEGs) during the differentiation of intramuscular and subcutaneous preadipocytes may help us to elucidate the differentially regulated mechanisms.

Previous study has compared the differences in gene expression profiles using microarray techniques between porcine intramuscular and subcutaneous mature adipocytes originating from isolated preadipocytes [8]. Proteomic studies of differential protein expression in undifferentiated and differentiated preadipocytes with respect to depots (omental, subcutaneous, and intramuscular) have indicated that preadipocytes originating from different fat depots have distinct adipogenic manners [2]. Solexa deep sequencing technique has been recently used to compare miRNA expression patterns in undifferentiated and differentiated porcine intramuscular and subcutaneous stromal vascular (SV) cells [9]. However, more experiments must be conducted to determine global DEGs during the process of primary porcine intramuscular and subcutaneous preadipocyte differentiation.

As a new preferred high-throughput gene expression quantification technique, RNA deep-sequencing methods such as Solexa RNA-Seq are widely used for genome-wide gene expression quantification analysis [10]. RNA-Seq refers to whole-transcriptome sequencing, wherein mRNA or cDNA is mechanically fragmented, resulting in overlapping short fragments that cover the entire transcriptome. And it verifies direct transcript profiling without compromise, thus allowing for more sensitive and accurate profiling of the transcriptome that more closely resembles the biology of the cell [10,11]. Additionally, recent studies have shown that RNA-Seq is more sensitive for low-expressed transcripts than traditional serial analysis of gene expression (SAGE) and microarray hybridization techniques [11,12].

In this study, SV cells were collected from postnatal porcine *longissimus dorsi* muscle (LM) and subcutaneous adipose tissue (SAT), and then induced to differentiate into adipocytes in vitro. RNA-Seq was used to screen DEGs between subcutaneous and skeletal intramuscular SV cells differentiation on days 0, 2, and 4. The main objective was to identify specially expressed genes and biological-function categories during the adipogenic differentiation of intramuscular SV cells.

## Materials and Methods

### Ethics Statement

 All animal procedures were performed according to pertinent guidelines (No. 5 Proclaimation of the Standing Committee of Hubei People’s Congress, P.R. China). Sample collection was approved by the ethics committee of Huazhong Agricultural University (approval permit number 30700571).

### Isolation of ASVC and MSVC

Three large white pigs (provided by Jingpin Pig farm of National Engineering Research Center on Farm Animals) were sacrificed at 3 days of age by intraperitoneal injection of pentobarbital sodium (50 mg/kg body weight) followed by exsanguination. SAT and LM were aseptically isolated and finely minced after removing all visible connective tissues. ASVC and MSVC were obtained based on previously reported methods [13,14] with some modifications. SAT and LM tissues were treated with digestion solution comprising 0.1% type-I and 0.2% type-II collagenase (Sigma), respectively, for 2 h at 37°C, followed by centrifugation of the digestion mixture at 1,000 × g for 8 min. Afterwards, the resulting mixture was filtered through 100 and 40 μm mesh filters and centrifuged for another 8 min at 1,000 × g to obtain SV cell pellets. These cell pellets were plated in proliferation medium comprising 90% DMEM and 10% fetal bovine serum (FBS), both from Gibco (Grand Island, NY, USA).

### Induction of ASVC and MSVC adipogenic differentiation and collection of cell samples

 After reaching confluence in proliferation medium (day 0), ASVC and MSVC were stimulated in adipogenic induction medium [DMEM comprising 10% FBS, 0.5 mM 3-isobutyl-1-methyxanthine (Sigma), 1μM dexamethasone (Sigma), and 10 μg/mL insulin (Sigma)] for 2 days, followed by 2 days of culture in medium (DMEM comprising 10% FBS and 10 μg/mL insulin). Cell samples from each time point (days 0, 2, and 4) were collected in triplicate during ASVC and MSVC adipogenic differentiation using Trizol reagent (Invitrogen), and then immediately dipped in liquid nitrogen, and stored at -80 °C until RNA extraction.

### Preparation of cDNA library and sequencing

Total cellular RNA was extracted from each sample using Trizol reagent (Invitrogen) according to the manufacturer’s instructions. The yield and purity of extracted RNA were assessed by measuring the absorbance (*A*) at 260 and 280 nm. RNA was used only when the *A_260_/A*_*280*_ ratio was > 1.8. RNA integrity was assessed using 1% agarose gel with RNA 6000 Nano Assay Kit and Agilent 2100 Bioanalyzer (Agilent, USA). The extracted total RNA was stored at -80°C for later use. For Illumina sequencing, the RNA samples from three independent biological replicates in each time point were pooled with the same amount of total RNA. Afterwards, 10 μg of total RNA from each pool was incubated with 10 U DNase i (Ambion) at 37 °C for 1 h, followed by a purification step to isolate poly (A) mRNA using a Micropoly (A) Purist TM mRNA purification kit (Ambion, USA). First-strand cDNA was synthesized from 10 μg of total RNA using GsuI-oligo dT primer and SuperScript II reverse transcriptase (Invitrogen, USA). After incubation at 42 °C for 1 h, the 5’-CAP structure of mRNA was oxidized by NaIO_4_ (Sigma) and ligated to biotin hydrazide, which was used to select complete mRNA/cDNA heterodimers by binding Dynal M280 beads (Invitrogen). Double-stranded cDNA was synthesized by primer extension using Ex Taq polymerase (TaKaRa). After second-strand cDNA synthesis, the polyA tails and 5’ adaptors were removed by GsuI digestion. To generate libraries for sequencing, double-stranded cDNA was fragmented to 300 bp to 500 bp sizes by sonication using a MISONIX Sonicator 3000 (QSonica, Newtown, CT, USA), followed by purification with Ampure beads (Agencourt, USA). Sequencing libraries (a total of six libraries) were prepared from the sheared cDNA using TruSeq™ DNA Sample Prep Kit-Set A (Illumina, USA). Sequencing was performed on an Illumina HiSeq 2000 sequencer using TruSeq Paired-End Cluster Kit v2.0 (Illumina, USA) and 200 cycle TruSeq SBS HS v2 Kit (Illumina, USA), generating 100 bp reads. The raw sequence data were deposited in the NCBI Sequence Read Archive under the accession number SRA092066.

### Real-time PCR validation

 To validate the sequencing results, eight DEGs were selected for further analysis by real-time PCR using the Lightcycler480 (Roche) and SYBR Premix Ex Taq Kit (Takara, Japan) according to the manufacturer’s instructions. Trizol reagent (Invitrogen, USA) was used to extract total RNA from Triplicate cell samples at each time point (ASVC and MASVC adipogenic differentiation on days 0, 2, and 4). First-strand cDNA was synthesized from 2 μg of total RNA with oligo(dT) and M-MLV Reverse Transcriptase (Toyobo, Japan), according to the manufacturer’s instructions. The primers used for the real-time PCR detection of selected genes are listed in Table S5. Endogenous β-actin mRNA was used as a reference for real-time PCR analysis. The relative expression levels were calculated by the 2^-ΔΔCt^ method [15]. Results were analyzed using a one-way analysis of variance (ANOVA) statistical test. All real-time PCR experiments were carried out on three biological replicates with three technical replicates for every sample.

### Analysis of DEGs and their expression profiles

Raw reads from each sequencing library were firstly cleaned using FASTX-Toolkit suite (http://hannonlab.cshl.edu/fastx_toolkit/) to remove adaptor sequences, reads with unknown sequences ‘N’ and low-quality sequences (the percentage of low-quality bases with a Phred quality score <20 was >50% in a read). The clean reads were mapped to reference sequences (NCBI FTP: ftp://ftp.ensembl.org/pub/release-68/fasta/ sus_scrofa/cdna) using Bowtie with default parameters [16]. The number of annotated clean reads for each gene was calculated and normalized to reads per kilobase per million (RPKM) [17]. Gene differential expression analysis was then conducted using MARS (MA-plot-based method with Random Sampling) model in DEGseq package between different time points during ASVC or MSVC adipogenic differentiation (ASVC differentiation on day 2 vs. day 0, ASVC differentiation on day 4 vs. day 0, ASVC differentiation on day 4 vs. day 2, MSVC differentiation on day 2 vs. day 0, MSVC differentiation on day 4 vs. day 0, and MSVC differentiation on day 4 vs. day 2). The “false discovery rate (FDR) ≤0.001 and the absolute value of log_2_fold change ≥1” were set as thresholds to judge the significance of gene expression difference [18].

Short Time-series Expression Miner (STEM) clustering method [19] was used to cluster the DEGs. The software is freely accessible over http://www.cs.cmu.edu/~jernst/st/. Each gene was assigned to the closest profile using a Pearson correlation based distance metric. To determine the significance level for a given transcriptome profile, a permutation-based test was used to quantify the expected number of genes that would be assigned to each profile [20].

### Gene Ontology and pathway analysis

 GO enrichment analysis with features corresponding to DEGs in each significant expression profile was performed using the online g:profiler [21] web server http://biit.cs.ut.ee/gprofiler/index.cgi, looking for significantly enriched GO terms in DEGs compared to the genome background. The significance level of GO term enrichment was set as FDR-adjusted *p* value less than 0.05. Pathway analysis was conducted using g:profiler web server, which was based on the Kyoto Encyclopedia of Genes and Genomes (KEGG) database. Pathways with statistically significance values (*p*<0.05) were selected.

### Transcription factor binding site (TFBS) analysis

 Analysis of TFBS enrichment across the genes based on the expression clusters was performed using the oPOSSUM Human Single Site Analysis package [22]. The oPOSSUM application is a web-accessible software system used to identify over-represented TFBS in sets of co-expressed genes generated from high-throughput sequencing methods [22]. oPOSSUM compares the occurrence of TFBS within a set of co-expressed genes with the background of all genes in the oPOSSUM database. The analysis was performed using the Human orthologous promoter sequence for each porcine gene. To detect common TFBS, the set of conserved regions was determined using thresholds of 0.4, and the matrix match threshold was 85% of the maximum match score. The promoters used in this study were from 2000 bp upstream of the transcription start site, which was selected because it agreed best with the tiling chip promoter areas [23]. Transcription factors were matched based on their corresponding binding sites, and over-presented transcription factors were considered to be significant at Z-score ≥10 and Fisher score ≥7.

## Results

### Illumina HiSeq 2000 sequencing and alignment to the reference genome

 To identify gene expression changes during porcine ASVC and MSVC differentiation and to compare whether the change is origin specific, an Illumina HiSeq 2000 sequencing experiment was carried out during the adipogenic differentiation of SV cells isolated from porcine LM and SAT. The following six cDNA libraries were sequenced: A0 (ASVC differentiation on day 0), A2 (ASVC differentiation on day 2), A4 (ASVC differentiation on day 4), M0 (MSVC differentiation on day 0), M2 (MSVC differentiation on day 2), and M4 (MSVC differentiation on day 4). High-quality clean reads were obtained from the six libraries (Table 1), Clean reads accounted for 97.85%, 98.43%, 98.57%, 98.52%, 98.28%, and 98.49% of the total reads, which meant that the raw reads from RNA-Seq were almost all high-quality reads.

**Table 1:** Summary of read numbers based on the RNA-Seq data.

	**A0**	**A2**	**A4**	**M0**	**M2**	**M4**
Total reads	5,821,523	5,799,965	5,724,965	5,671,635	5,614,635	5,663,565
Clean reads	5,696,411	5,708,849	5,648,758	5,587,880	5,518,231	5,577,959
Ratio	97.85%	98.43%	98.67%	98.52%	98.28%	98.49%
Mean length of reads	100	100	100	100	100	100

A0, A2, A4 respectively represented ASVC adipogenic differentiation on days 0, 2, and 4; M0, M2, M4 respectively represented MSVC adipogenic differentiation on days 0, 2, and 4.

### Global analysis of gene expression

 The distribution of read expression was used to evaluate the normality of our RNA-Seq data. As shown in Figure S1, the distribution of distinct reads over different read abundance categories showed similar patterns for all six RNA-Seq libraries. The similarity distribution had a comparable pattern, with more than 50% of the sequences having a similarity >70%, which indicated the high accuracy of annotated genes through the mapping reads.

A total of 20,353 genes, ranging from 100 to ≥2000 bp, were detected during ASVC and MSVC differentiation. 8,333 (40.94%) genes were more than 1000 bp in length. The length of 3,635 (17.86%) genes ranged from 1500 to 2000 bp, 4,029 (19.80%) ranged from 1000 to 1500 bp, 3,632 (17.84%) ranged from 500 to 1000 bp, and 724 (3.56%) ranged from 100 to 500 bp (Table S1). 

**Figure 1:**
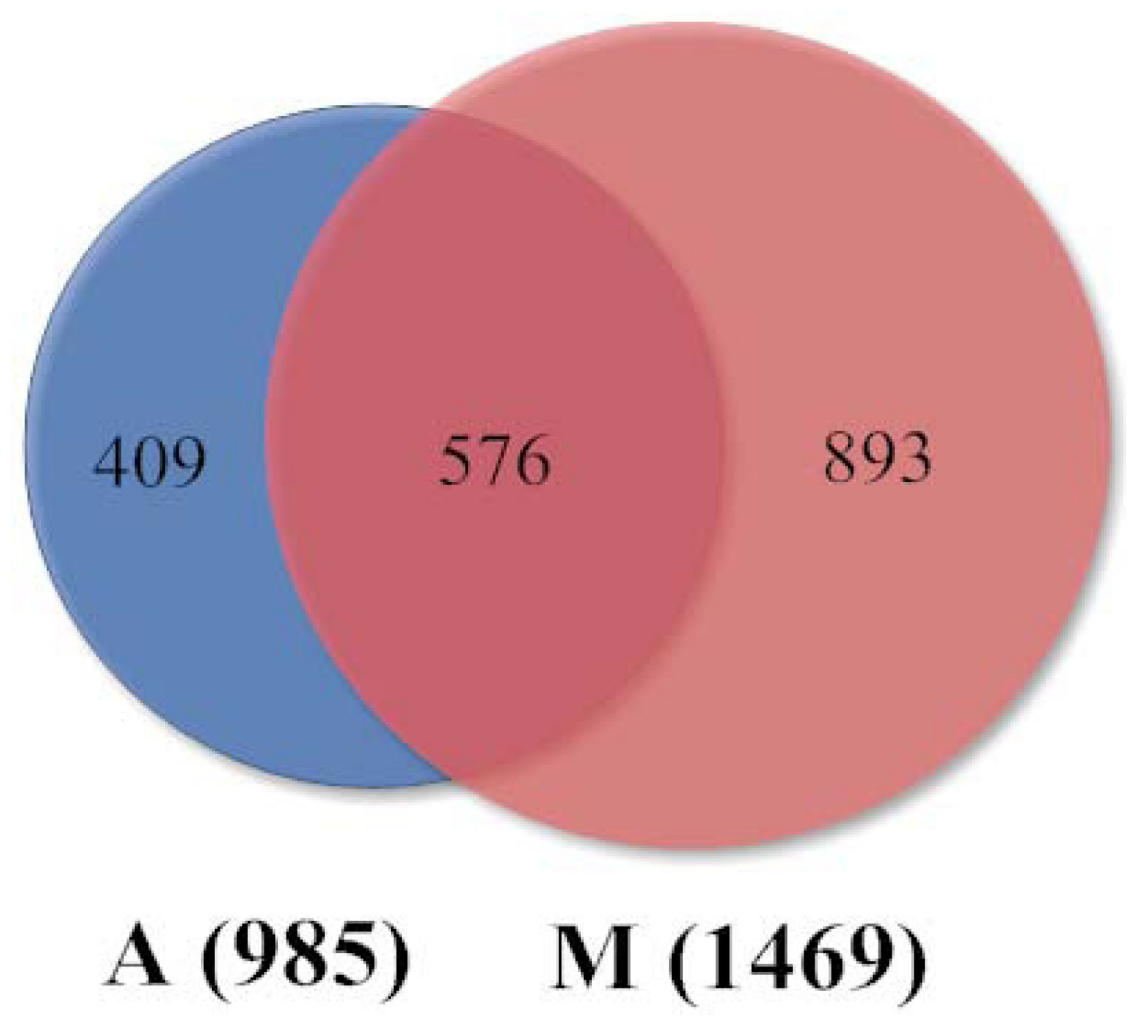
Venn diagram showing the DEGs during ASVC and MSVC adipogenic differentiation. A, ASVC adipogenic differentiation; M, MSVC adipogenic differentiation. Among these DEGs, 985 are expressed in A, 1469 are expressed in M, 576 are co-expressed in A and M. The numbers of specific DEGs are 409 (A) and 893 (M).

To identify genes with significant changes in expression levels during ASVC and MSVC differentiation, six comparisons of gene expression from the three time points during ASVC adipogenic differentiation (A2 vs. A0, A4 vs. A0, and A4 vs. A2) and the three time points during MSVC adipogenic differentiation (M2 vs. M0, M4 vs. M0, and M4 vs. M2) were investigated. Genes were considered to be DEGs only when the fold-change in abundance for at least one comparison was more than or equal to two fold (the absolute value of log_2_fold change ≥1) with FDR ≤0.001. The DEGs are summarized in Figure 1. A total of 1,878 DEGs were detected for both ASVC and MSVC differentiation, 985 DEGs were expressed in ASVC differentiation and 1469 DEGs were expressed in MSVC differentiation. Among 1,878 DEGs, 576 were co-expressed in ASVC and MSVC differentiation, 409 DEGs were specific for ASVC differentiation, and 893 DEGs were specific for MSVC differentiation. DEGs in each cluster were shown in Table S2.

### Validation of RNA-Seq-based gene expression

**Figure 2:**
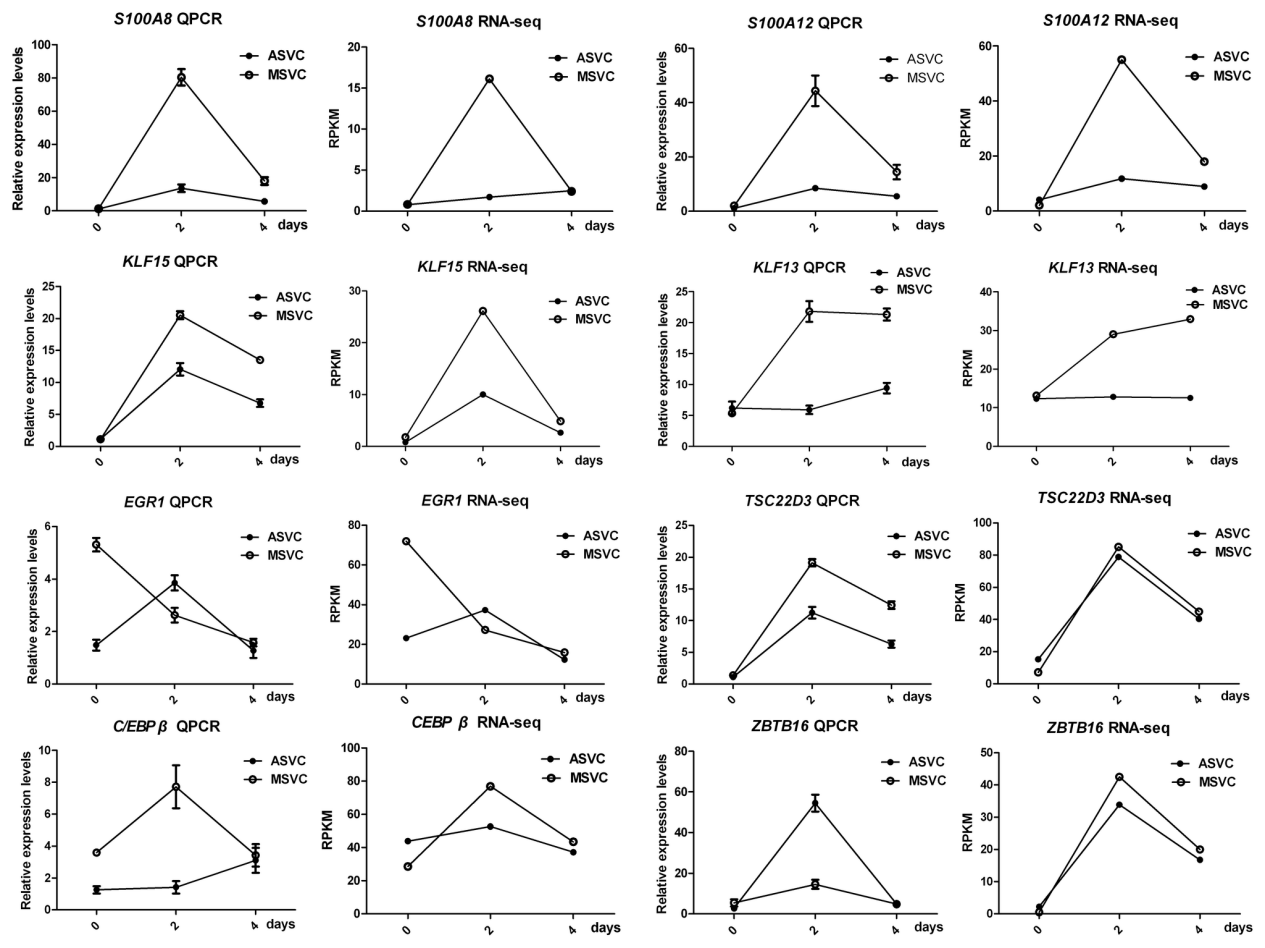
Validation of the RNA-Seq data by Q-PCR analysis. Q-PCR and RNA-Seq results of eight genes (*S100A8, S100A12, KLF15, KLF13, EGR1, TSC22D3, C/EBPβ*, and *ZBTB16*) during ASVC and MSVC differentiation. Relative expression levels of genes were calculated based on the mean value from three pigs by using the comparative Ct method.

 To validate the results of RNA-Seq, eight genes are chosen for real-time quantitative RT-PCR (Q-PCR) analysis. The results of Q-PCR are shown in Table S6. Figure 2 displays the results of gene expression patterns derived from RNA-Seq and Q-PCR experiments during ASVC and MSVC adipogenic differentiation. For six of the eight genes, i.e., *S100A8, S100A12*, *KLF13, KLF15, EGR1*, and *TSC22D3*, the Q-PCR expression profiles completely agreed with the RNA-Seq data, whereas for the remaining two genes *C/EBPβ and ZBTB16*, the extent of the changes in gene expression levels as measured by the two methods did not exactly match (Figure 2). These data suggested that the results of RNA-Seq analysis were reliable indicators of overall changes in gene expression.

### Clustering of DEGs during ASVC and MSVC adipogenic differentiation processes

**Figure 3:**
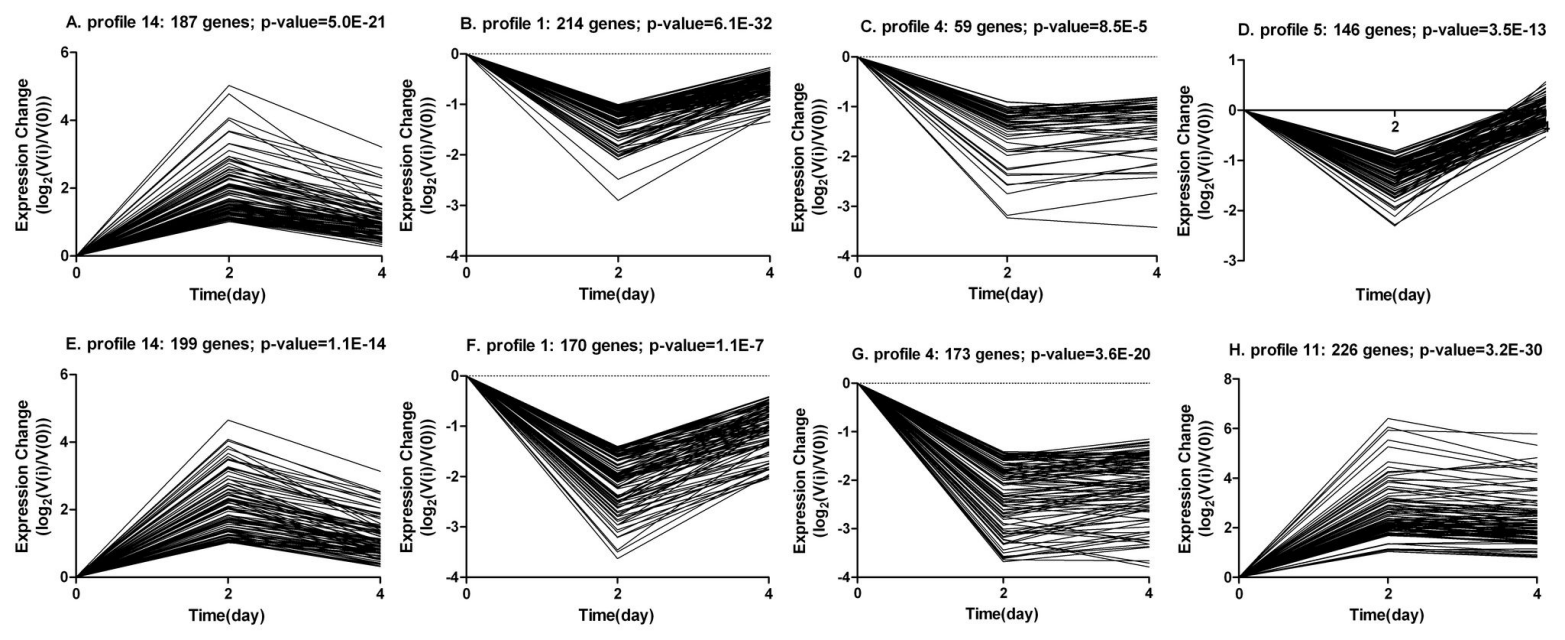
STEM clustering on DEGs during ASVC and MSVC adipogenic differentiation. Significant gene expression profiles resulting from c = 2 and m = 50 (c indicates maximum unit change in model profiles between time points, m indicates maximum number of model profiles) are displayed as time course plots of log_2_ gene expression ratios on day 2 or 4 vs. day 0. The number of genes and *p* value in each profile are shown. Time is shown in days. A–D, four significant gene expression profiles of DEGs during ASVC differentiation; E–H, four significant gene expression profiles of DEGs during MSVC differentiation.

The expression profiles of DEGs were determined by cluster analysis based on the STEM platform with default parameters c = 2 and m = 50, where c is the maximum unit change in model profiles between time points and m is the maximum number of model profiles. Figure 3 shows that 4 gene expression profiles (A–D) of DEGs during ASVC differentiation and 4 gene expression profiles (E–H) of DEGs during MSVC differentiation were significant out of 50 candidate profiles. Gene expression profiles 14, 1, and 4 were found to be involved in both ASVC and MSVC differentiation. Moreover, DEGs in Profile 14 were positively modulated at the early (day 2) and middle (day 4) differentiation stages, whereas DEGs in profile 1 and 4 were negatively modulated at the early and middle stages of differentiation. Among the four significant adipose profiles (A–D), profile 1 showed the most abundant gene expression, which comprised 214 genes with a p-value of 6.1 × 10^-32^. The expression pattern of profile 1 showed downregulation from day 0 to day 2, followed by up-regulated to day 4. The second most abundant profile was profile 14, which comprised 187 genes and *p* =5.0 × 10^-21^, which showed upregulation from day 0 to day 2, followed by down-regulated to day 4. Among the four significant muscle profiles (E–H), profile 11 was the most abundant and comprised 226 genes that were up-regulated from day 0 to day 2, and then maintained high expression level. Profile 14 was the second most abundant profile comprising 199 genes and *p* =1.1 × 10^-14^. With the similar expression pattern between profile 11 and profile 14, the STEM tool clustered them into one cluster (Figure 3).

### Functional analysis of DEGs involved in the significant gene expression profiles

**Figure 4:**
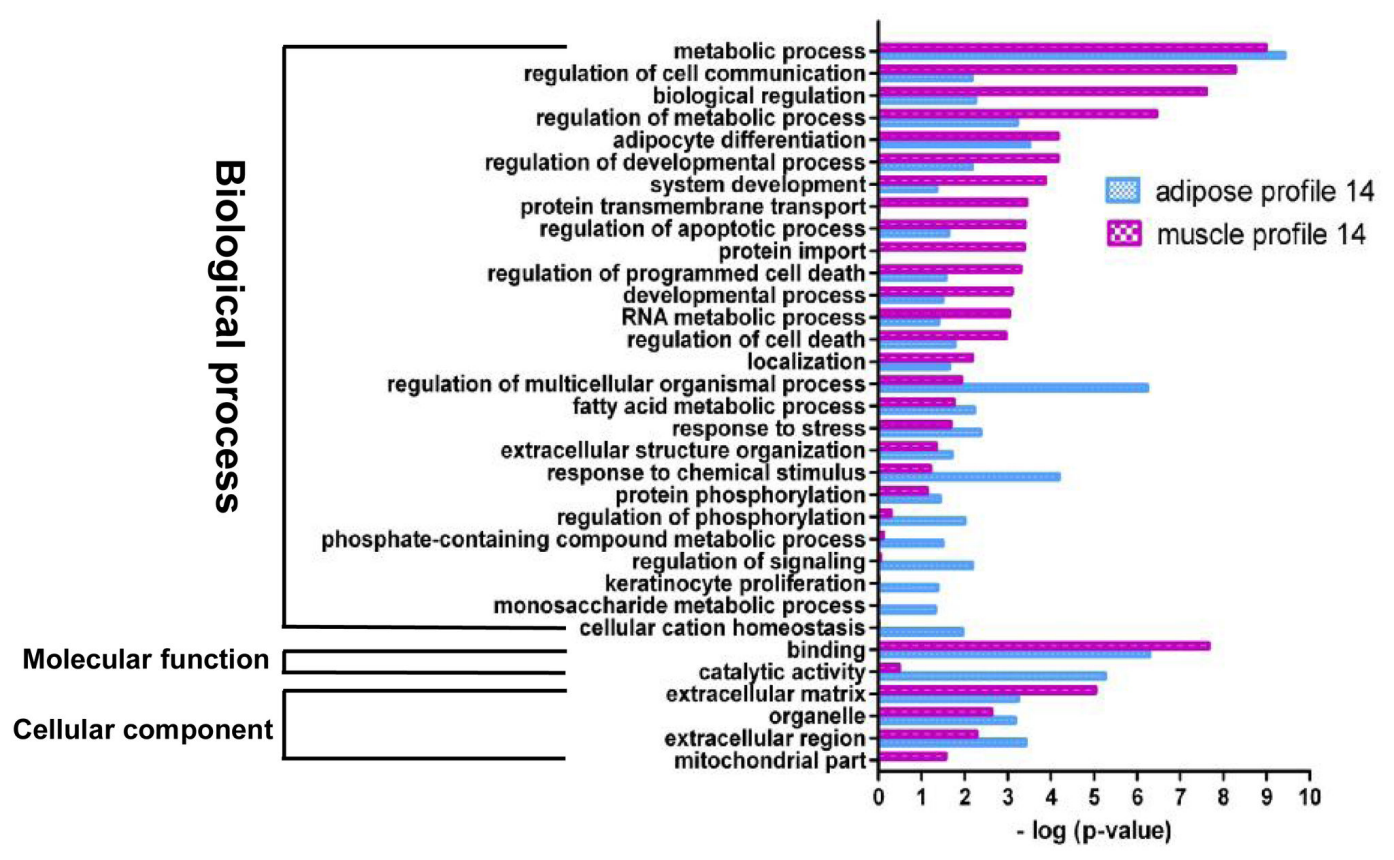
GO functional enrichment analysis of DEGs in adipose and muscle profile 14. The results are summarized in the following three main categories: biological process, molecular function, and cellular component. The y-axis indicates functional groups. The x-axis indicates –log (*p* value).

**Figure 5:**
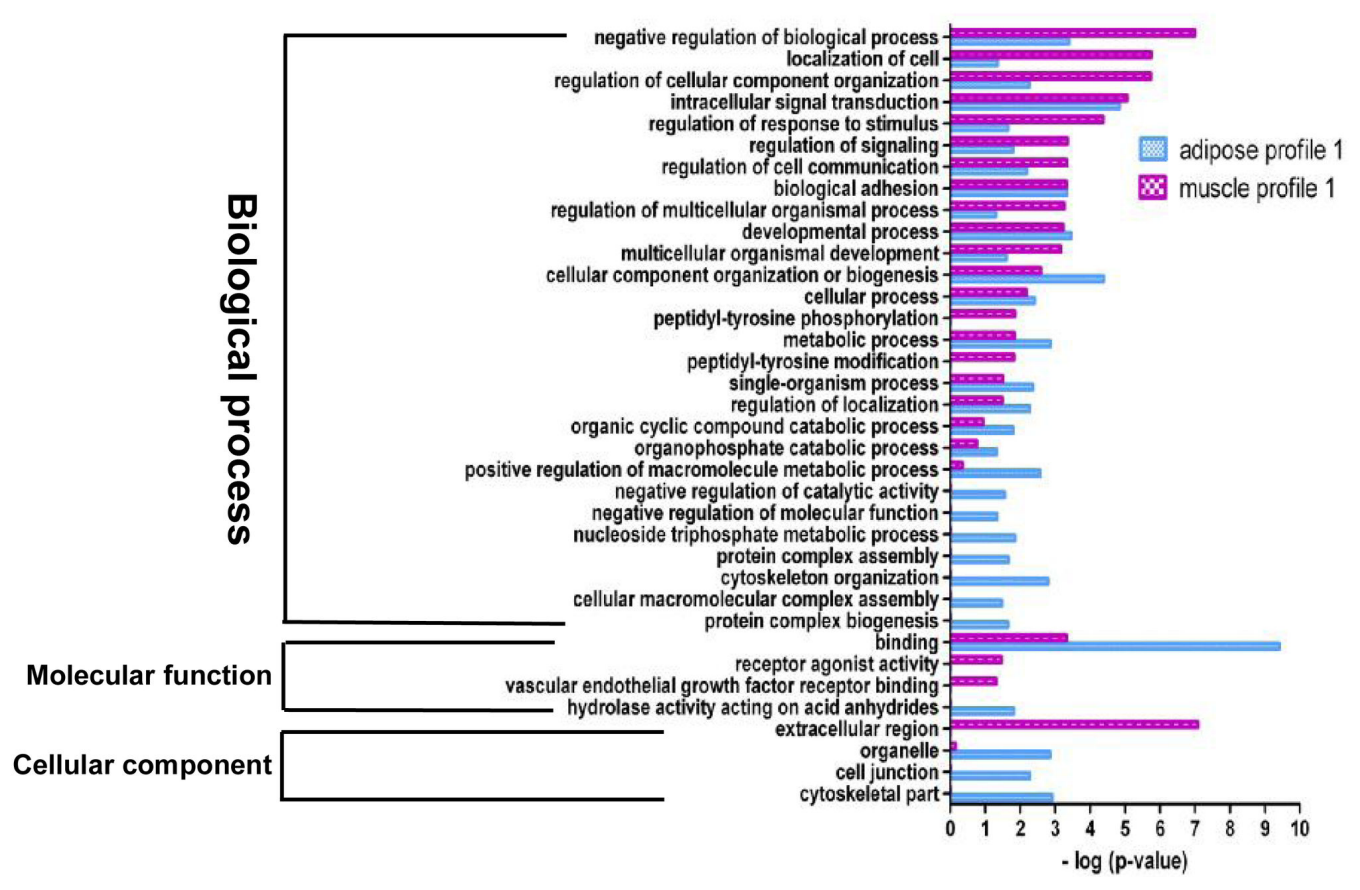
GO functional enrichment analysis of DEGs in adipose and muscle profile 1. The results are summarized in the following three main categories: biological process, molecular function, and cellular component. The y-axis indicates functional groups. The x-axis indicates –log (*p* value).

**Figure 6:**
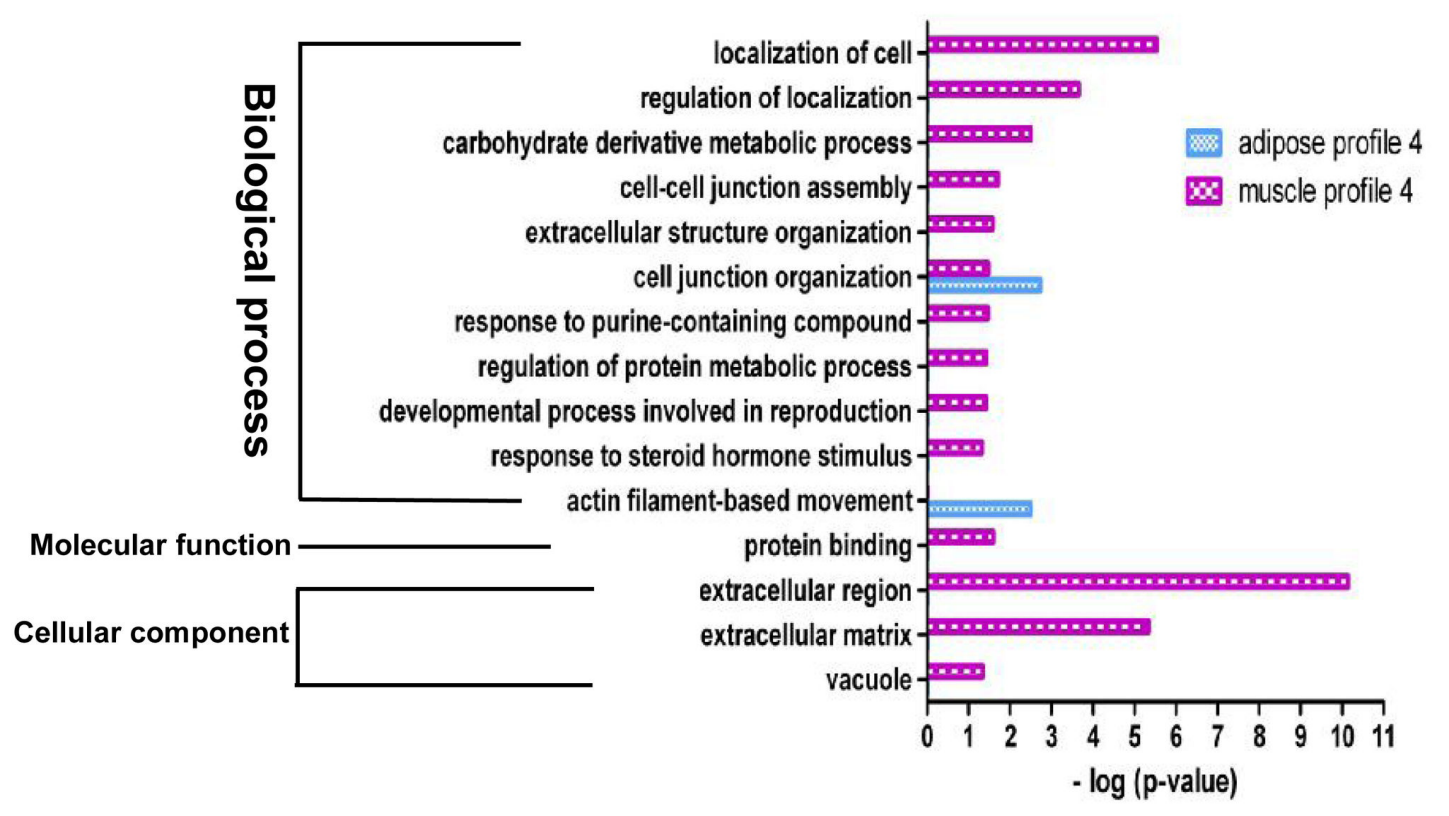
GO functional enrichment analysis of DEGs in adipose and muscle profile 4. The results are summarized in the following three main categories: biological process, molecular function, and cellular component. The y-axis indicates functional groups. The x-axis indicates –log (*p* value).

**Figure 7:**
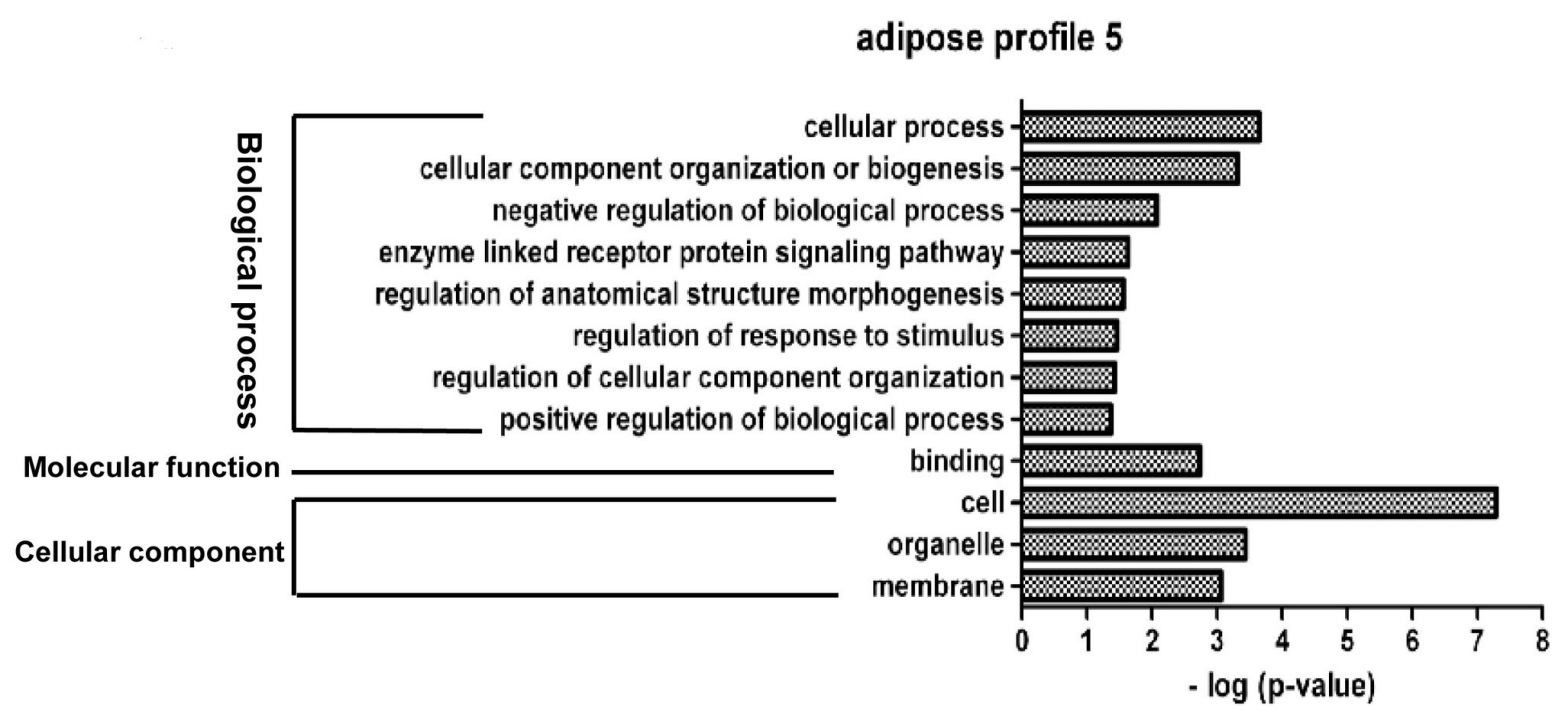
GO functional enrichment analysis of DEGs in adipose profile 5. The results are summarized in the following three main categories: biological process, molecular function and cellular component. The y-axis indicates functional groups. The x-axis indicates –log (*p* value).

**Figure 8:**
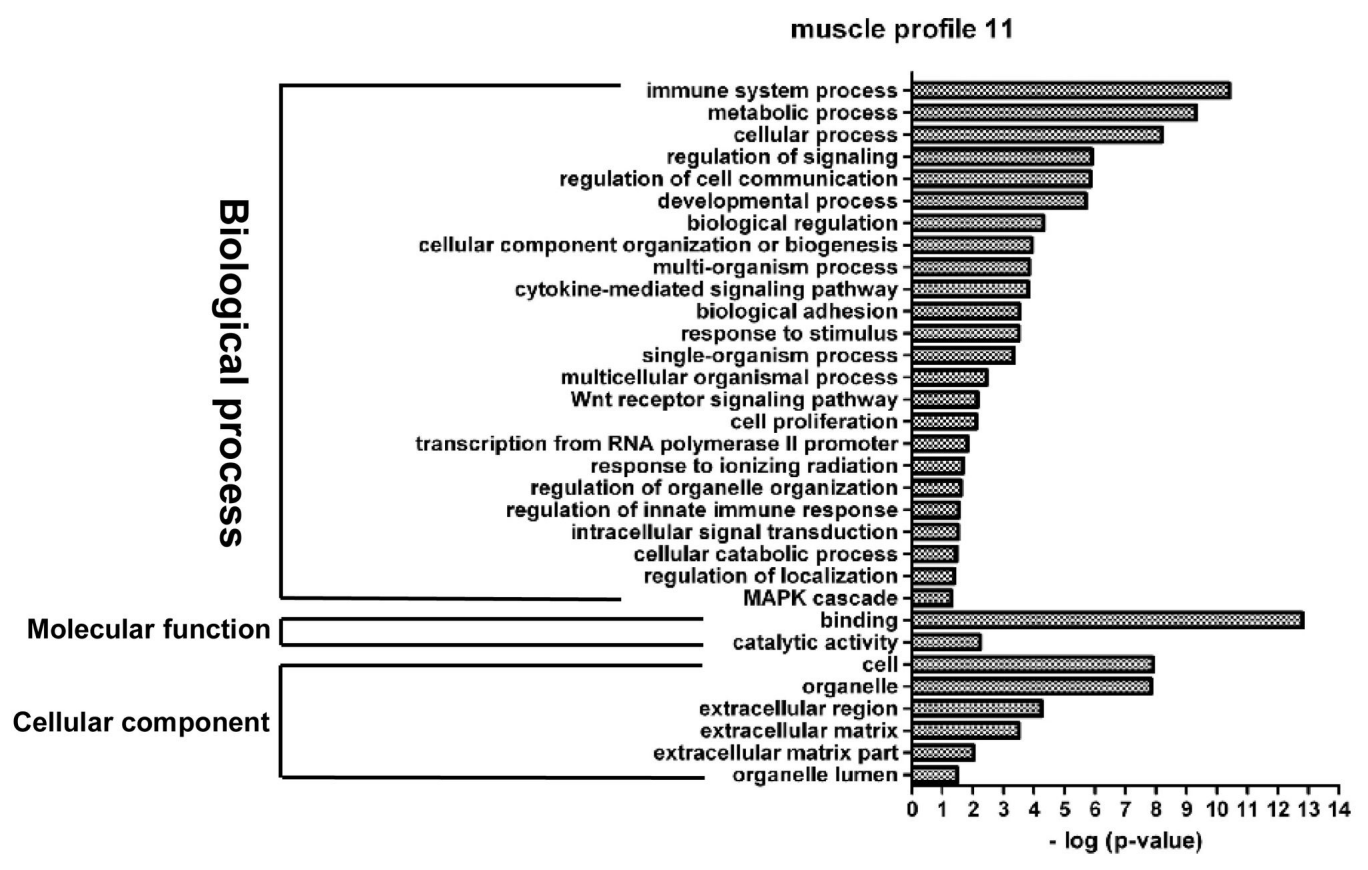
GO functional enrichment analysis of DEGs in muscle profile 11. The results are summarized in the following three main categories: biological process, molecular function and cellular component. The y-axis indicates functional groups. The x-axis indicates –log (*p* value).

GO analysis were preformed to further understand the biological functions of the genes within significant gene expression profiles. Significant GO categories with *p* <0.05 (Figure 4–8) were selected. Results showed that DEGs related to adipocyte differentiation and fatty acid metabolism were significantly enriched in both adipose and muscle gene expression profile 14. Additionally, several fundamental biological processes were found to be notably enriched in both adipose and muscle gene expression profile 14, such as metabolic process, regulation of cell communication, biological regulation and regulation of developmental process (Figure 4). The abovementioned fundamental biological processes may be essential for both ASVC and MSVC adipogenic differentiation. Some functional categories were found to be specifically enriched in muscle profile 14, such as protein transmembrane transport and protein import. Cellular component function analysis also indicated that extracellular matrix (ECM) and extracellular region terms were prominently enriched in both adipose and muscle gene expression profile 14, suggesting that the extracellular environment of SV cells from distinct fat depots (ASVC and MSVC) may play an important role in the adipogenic differentiation of SV cells (Figure 4). 

The genes in adipose expression profile 1 were mainly clustered into the following functional groups: intracellular signal transduction, cellular component organization or biogenesis, negative regulation of biological process, binding (Figure 5). Figure 5 also shows that the genes in muscle profile 1 were mainly clustered into the following functional groups: negative regulation of biological process, localization of cell, regulation of cellular component organization, regulation of response to stimulus. Adipose profile 4 consisted of only 59 genes, which were clustered into two significant GO categories, namely, cell junction organization, actin filament-based movement (Figure 6). However, muscle profile 4 comprised 173 genes that were clustered into 14 significant GO categories (Figure 6), which were predominant cell localization, regulation of localization, carbohydrate derivative metabolic process, extracellular region and ECM.

 Profile 5 is an adipose-specific significant gene expression profile, wherein 146 genes were mainly clustered into functional groups, i.e., cellular process, cellular component organization or biogenesis, enzyme linked receptor protein signaling pathway, and binding processes (Figure 7). Profile 11 is a muscle-specific gene expression profile, wherein 226 genes were clustered into 32 significant GO categories, and the main functional groups included immune system process, metabolic process, cellular process, regulation of signaling, and regulation of cell communication (Figure 8). 

**Figure 9:**
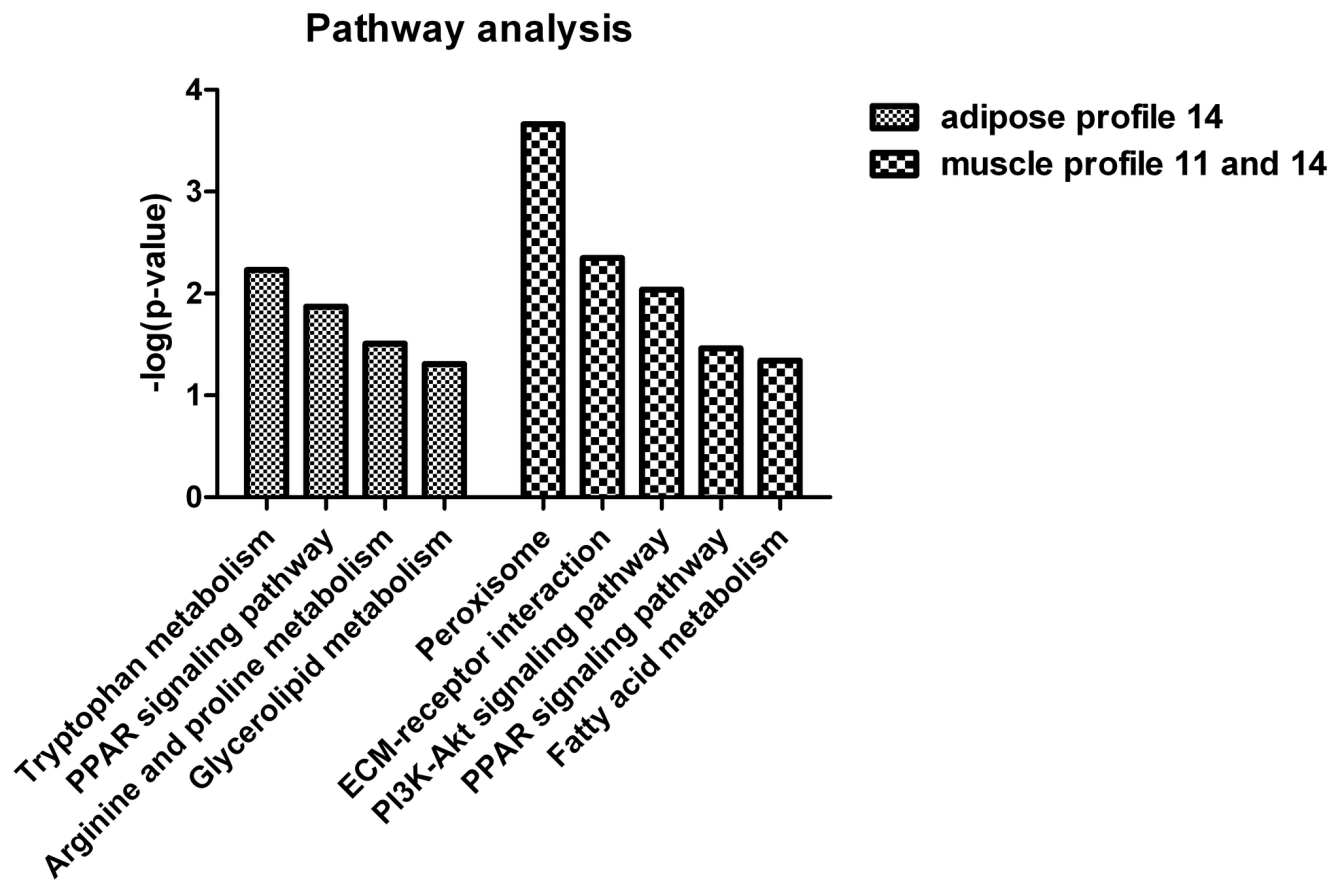
KEGG pathway enrichment analysis of DEGs in adipose profile 14 and muscle profiles 11 and 14. Results show the significant enrichment pathways in adipose profile 14 and muscle profiles 11 and 14 respectively. The x-axis indicates functional pathways. The y-axis indicates –log (*p* value).

The genes related to adipocyte differentiation and fatty acid metabolism categories are shown in gene expression profile 14. To further understand the biological functions of the genes related to adipocyte differentiation and fatty acid metabolism, the genes in adipose profile 14 or muscle profiles 11 and 14 (grouped into a single cluster by STEM) were mapped to terms in the KEGG database and compared with the human transcriptome background (Figure 9). The results showed that genes within adipose profile 14 were significantly enriched in tryptophan metabolism, PPAR signaling pathway, arginine and proline metabolism, as well as glycerolipid metabolism (*p*<0.05). Among these signaling pathways, PPAR signaling pathway was significantly enriched in both adipose profile 14 and muscle profiles 11 and 14. Additionally, genes in muscle profiles 11 and 14 were specifically significantly enriched in peroxisome, ECM-receptor interaction, PI3K-Akt signaling pathway and fatty acid metabolism signaling pathways comparing with the pathway analysis of genes in adipose profile 14 (*p*<0.05). These results suggest distinct regulatory mechanisms operating between the adipose and muscle-derived SV cells adipogenic.

### Identification of over-represented transcription factor binding sites in sets of co-expressed genes

 To identify the specific transcription factors that regulate MSVC adipogenic differentiation, oPOSSUM was used to identify over-presented TFBS in the promoters of sets of co-expressed genes from adipose profile 14 and muscle profiles 11 and 14 (Table 2). The current analysis reveal that potential SRY, CEBPA, FOXA1, TBP, and HLF binding sites were significantly enriched in the promoter regions of genes in adipose profile 14. A total of 87, 76, 73, 67, and 37 genes contained respective binding sites for transcription factors SRY, CEBPA, FOXA1, TBP, and HLF within their promoters. In addition, only the two transcription factors NR3C4 and NR3C1, which comprised significant over-represented TFBS, were found within the promoters of genes from muscle profiles 11 and 14. Fifteen target genes for transcription factor NR3C4 and 37 target genes for transcription factor NR3C1 were found in the current analysis. 

**Table 2:** oPOSSUM analysis of over-represented transcription factor binding sites.

**Gene expression profile**	**TF^A^**	**Family**	**No.^B^**	**Z-score**	**Fisher score**
Adipose profile 14	TBP	TATA-binding	67	16.25	14.26
	SRY	High Mobility Group	87	13.88	10.58
	HLF	Leucine Zipper	37	12.81	12.29
	FOXA1	Forkhead	73	11.99	7.90
	CEBPA	Leucine Zipper	76	11.11	10.89
Muscle profile 11 and 14	NR3C4	Hormone-nuclear Receptor	15	24.17	14.55
	NR3C1	Hormone-nuclear Receptor	31	11.07	10.60

(A) TF, transcription factor; (B) Number of target genes.

## Discussion

### DEGs during ASVC and MSVC differentiation

SV cells undergoing adipogenic differentiation exhibited well-characterized morphological changes reflected in the cell transcriptome. During the adipogenic differentiation of SV cells, there is an exquisitely coordinated alteration in gene expression that regulates the conversion of preadipocytes into mature adipocytes. During 3T3-L1 preadipocyte differentiation, a substantial number of genes have been identified as regulated in a differentiation-dependent manner [24]. In the current study, the same induction conditions were used to stimulate ASVC and MSVC adipogenic differentiation, and RNA-Seq analysis was conducted to create a comprehensive view of the global DEGs during the adipogenic differentiation of ASVC and MSVC. Results showed different number of DEGs and specifically expressed genes during the adipogenic differentiation of ASCV and MSVC (Figure 1). Moreover, a higher number of total DEGs and specifically expressed genes existed during MSVC adipogenic differentiation. These results can be explained as follows: First, cell types involved in ASVC or MSVC were different, primary ASVC included preadipocytes, adipoblasts, adipose stem cells, endothelial cells, pericytes, and blood cells [25], whereas primary MSVC included satellite cells, mesenchymal stem cells, and myogenic cells other than preadipocytes [26,27]. The different cell types may be extrinsic factors affecting the different number of DEGs and specifically expressed genes during the adipogenic differentiation of ASCV and MSVC. Second, preadipocytes derived from different fat depots appear to have distinct adipogenic potential [5], which may be an intrinsic factor causing the different number of DEGs and specifically expressed genes during the adipogenic differentiation of ASCV and MSVC. 

### Clustering the patterns of DEGs during ASVC and MSVC adipogenic differentiation

 Many distinct gene expression patterns can be observed during adipogenic differentiation. Some genes such as the transcription factors GATA2 and GATA3 are transiently induced and expressed at early stages and control preadipocyte to adipocyte transition [28], On the other hand, some genes are constitutively activated, i.e., adipogenic transcription factors, peroxisome proliferator activated receptor-γ (PPARγ), and CCAAT-enhancer binding protein-α (C/EBPα), which are involved in regulating terminal differentiation [29]. Therefore, some distinct gene expression profiles were present during SV cells adipogenic differentiation. In this study, four significant gene expression profiles (profiles 1, 4, 5, and 14) in ASVC differentiation and four significant gene expression profiles (profiles 1, 4, 11, and 14) in MSVC differentiation (Figure 3) are observed. Interestingly, most significant gene expression profiles were substantially enriched in both ASVC and MSVC differentiation, such as gene expression profiles 1, 4, and 14. GO functional analysis demonstrated that some biological categories were involved in both ASVC and MSVC adipogenic differentiation (Figure 4-8). The genes were involved in these same biological categories with similar expression patterns. Therefore, most significant gene expression profiles were present in both ASVC and MSVC differentiation processes. For example, fatty acid metabolism was significantly enriched during both ASVC and MSVC differentiation processes and comprised the same genes *LPL, TRIB3*, and *PPARG* with similar expression patterns. The genes *RGS2, CEBPA, PPARG*, and *IGF1* were all related to adipocyte differentiation and clustered into the same profile (Table S3, and S4). However, SV cells derived from porcine subcutaneous and IMF tissue have distinct adipogenic potentials, and the regulation manners of ASVC and MSVC adipogenic differentiation were different [8]. Therefore, specific gene expression profiles were observed in the ASVC or MSVC adipogenic differentiation process. In this study, significant gene expression pattern profile 5 was specific to ASVC adipogenic differentiation, whereas the significant gene expression profile 11 was specific to MSVC adipogenic differentiation.

### Functional analysis of DEGs involved in the significant profiles

 Adipocyte differentiation requires the cells to process a variety of combinatorial biological groups during the decision to undergo differentiation [30]. Differentiation itself is characterized by changes in cell morphology and is regulated by complex molecular events controlled by signaling from hormones [31]. Accordingly, GO term analysis was used to explore the function of DEGs involved in the significant profiles. As expected, adipocyte differentiation was found to be significantly enriched in gene expression profile 14 of DEGs during both ASVC and MSVC adipogenic differentiation, which also comprised some general functional groups including metabolic process, biological regulation, regulation of metabolic process, and regulation of developmental process (Figure 4). These general functional groups may be essential to the conversion of preadipocytes into adipocytes. Moreover, cellular component function analysis indicated that ECM and extracellular region terms were significantly enriched in both adipose and muscle gene expression profile 14 (Figure 4), which suggested that the extracellular environment of SV cells originating from distinct fat depots (ASVC and MSVC) may have markedly affected the adipogenic differentiation of SV cells. To clearly understand the regulation mechanism of ASVC and MSVC differentiation, KEGG pathway analysis was used to explore the signaling pathways of DEGs involved in adipose profile 14 and muscle profiles 11 and 14. Several well-known pathways related to adipocyte differentiation were found to be involved in ASVC and MSVC adipogenic differentiation, such as PPAR signaling pathway, glycerolipid metabolism and fatty acid metabolism pathway (Figure 9). 

 KEGG analysis provided the first demonstration that peroxisome may specifically contribute to MSVC differentiation. Peroxisomes have long been established to play a central role in regulating various metabolic activities in mammalian cells, and the peroxisome pathway controls the fatty acid-oxidation and reactive antioxidant systems [32], which is consistent with GO terms fatty acid oxidation and response to stress being significantly enriched in muscle gene expression profiles 11 and 14. To our knowledge, no work has studied the involvement of the peroxisome pathway in regulating adipocyte differentiation. The ECM-receptor interaction pathway can directly or indirectly influence cellular activities such as adhesion and migration [33]. The change in shape of fibroblastic preadipocytes to rounded, mature adipocytes is accompanied by changes in cytoskeletal organization and contacts with the ECM [34]. Notably, the KEGG pathway analysis showed that the ECM-receptor interaction pathway was significantly enriched and specific to MSVC adipogenic differentiation. Therefore, we speculate that the ECM-receptor interaction pathway may participate in MSVC differentiation process. The intracellular PI3K-Akt signaling pathway is involved in the regulation of many cellular processes [35]. In particular, several lines of evidence have implicated the PI3K-Akt signaling pathway as a positive regulator of adipocyte differentiation. Disruption of PI3K function by pharmacological inhibitors [36] or dominant negative mutations [37] abolishes adipocyte differentiation from preadipocytes. These previous studies have indicated that the PI3K-Akt signaling pathway is essential for the preadipocyte differentiation. A recent study has demonstrated that the insulin-PI3K-Akt signaling pathway is significantly enriched for targets of IMF-specific miRNAs other than subcutaneous-specific miRNAs [9]. In the present study, the PI3K-Akt signaling pathway was specifically enriched significantly for DEGs involved in muscle profiles 11 and 14 (Figure 9), consistent with the results of previous studies. Thus, the PI3K-Akt signaling pathway may be an important signaling pathway for specifically regulating MSVC adipogenic differentiation.

### Identification of over-represented transcription factor binding sites in sets of co-expressed genes

 Transcription factors play important roles in the regulation of adipocyte differentiation. Significant advances toward elucidating the regulatory mechanisms involved in adipocyte differentiation have been carried out mostly by identifying transcription factors that contribute to the adipogenic process [38]. In order to identify the over-represented transcription factors that may participate in ASVC and MSVC adipogenic differentiation, we used the web-based tool oPOSSUM to identify over-represented TFBS in the promoters of sets of co-expressed genes in adipose gene expression profile 14 and muscle gene expression profiles 11 and 14. TFBS analysis results showed that potential TBP, SRY, HLF, FOXA1, and CEBPA binding sites were significantly enriched in the promoter regions of genes in adipose gene expression profile 14. Transcription factors CEBPA and FOXA1 have been previously demonstrated to play important roles in adipocyte differentiation [38,39]. C/EBP transcription factors were the first family of transcription factors shown to play a critical role in the adipocyte differentiation in vitro [38]. CEBPA is well known to be the primary transcription factor that mediates adipogenesis, and co-expressed genes in adipose profile 14 comprise *CEBPA* gene. Thus, the transcription factor CEBPA may participate in the regulation of ASVC adipogenic differentiation. Additionally, the transcription factor FOXA1 binding site was significantly enriched in the promoter regions of genes in adipose gene expression profile 14. FOXA1 is a member of the Forkhead/winged helix transcription factor family that has been proven to participate in the regulation of differentiation, metabolism, and developmental processes [40]. Previous studies found that FOXA1 was expressed in preadipocytes, and up-regulated at the early phase of adipogenesis. Moreover, *FOXA1* has been found to be a novel target gene of CEBPB, suppressing lipid accumulation with the down-regulation of the expression of adipogenic gene expression in adipocytes [39]. In the current analysis, 73 genes contained binding sites for FOXA1 within their promoters, indicating that the transcription factor FOXA1 may be associated with ASVC adipogenic differentiation. So far, very little work has yet been conducted to investigate the roles of transcription factors TBP, SRY, and HLF in regulating adipocyte differentiation. Further studies are needed to determine whether these transcription factors play important roles in ASVC adipogenic differentiation. 

Additionally, the binding sites of transcription factors NR3C4 and NR3C1 were found to be significantly enriched in the promoter regions of genes within muscle gene expression profiles 11 and 14. The two transcription factors are both nuclear hormone receptors. NR3C4, also known as androgen receptor, is a DNA-binding transcription factor that regulates gene expression [41]. NR3C4 is activated by binding of either of the androgenic hormones testosterone or dihydrotestosterone, which inhibit adipocyte differentiation in vitro through an NR3C4-mediated nuclear translocation of β-catenin and activation of downstream Wnt signaling [42]. In the present study, 15 genes in muscle profiles 11 and 14 comprised NR3C4 binding sites within their promoters. Among the 15 target genes, *ZBTB16*, *NFIA*, and *KLF15* have been demonstrated to positively regulate adipogenic differentiation [43-45]. Therefore, the transcription factor NR3C4 may be involved in MSVC adipogenic differentiation. NR3C1, also known as glucocorticoid receptor, can function both as a transcription factor that binds to glucocorticoid response elements in the promoters of glucocorticoid responsive genes to activate their transcription and as a regulator of other transcription factors. Glucocorticoids are known to be powerful regulators of adipocyte differentiation [46], and glucocorticoid response is mediated through the intracellular glucocorticoid receptor (NR3C1) to regulate adipocyte differentiation [47]. NR3C1 plays important roles in adipocyte differentiation. In the current analysis, 31 genes within muscle profiles 11 and 14 were detected to contain binding sites for NR3C1 within their promoters, indicating that NR3C1 may participate in the regulation of MSVC adipogenic differentiation. 

## Conclusions

This is a novel study comparing DEGs of ASVC and MSVC during adipogenic differentiation. The study produced abundant data for the analysis of ASVC and MSVC adipogenic differentiation. A lot of DEGs were found to be involved in both ASVC and MSVC adipogenic differentiation, and some fat-depot-specific DEGs were found during ASVC and MSVC adipogenic differentiation. GO analysis results indicated that DEGs related to adipocyte differentiation in ASVC and MSVC differentiation were clustered into the same gene expression profile (profile 14). Further function analysis demonstrated that DEGs in adipose and muscle profile 14 were regulated by distinct transcription factors and regulated ASVC and MSVC adipogenic differentiation through the distinct signaling pathways. Additionally, the extracellular environment of SV cells from distinct fat depots (ASVC and MSVC) may play an important role in the adipogenic differentiation of SV cells. The present findings establish the groundwork and provide new clues for uncovering the molecular mechanisms underlying IMF deposition in pigs. However, the RNA samples from three independent biological replicates in each time point were pooled with equal quality for Illumina HiSeq 2000 sequencing in present study, the deep sequencing results may need to be validated with more biological replicates. 

## Supporting Information

Figure S1 **Percentage coverage representing the percentage of genes expressed in each of the six samples mapping in the pig genome.** A0, ASVC differentiation on day 0; A2, ASVC differentiation on day 2; A4, ASVC differentiation on day 4; M0, MSVC differentiation on day 0; M2, MSVC differentiation on day 2; M4, MSVC differentiation on day 4. Gene coverage is the percentage of a gene covered by reads. This value is equal to the ratio of the base number in a gene covered by mapping reads to the total bases number of that gene. The distribution of distinct reads over different read abundance categories show similar patterns for all six RNA-Seq libraries.
(TIF)

Table S1 **Size distribution of gene sequences detected in ASVC and MSVC differentiation using RNA-Seq.**
(DOCX)

Table S2 **Detailed information on DEGs during ASVC and MSVC adipogenic differentiation.** We used FDR < 0.001 and the value of log_2_ Ratio ≥ 1or ≤ -1 as the threshold to judge the significant of gene expression difference. In order to calculate the log_2_ Ratio and FDR, we used RPKM value of 0.001 instead of 0 for genes that do not express in the samples. A0, A2, A4 respectively represented ASVC adipogenic differentiation on days 0, 2, and 4; M0, M2, M4 respectively represented MSVC adipogenic differentiation on days 0, 2, and 4.
(XLSX)

Table S3 **Detailed information on DEGs within four significant expression profiles during ASVC differentiation.** Four gene expression profiles (profiles 14, 1, 5, and 4) were significantly enriched in ASVC differentiation. DEGs in each gene expression profile were shown. The gene expression levels were normalized by the log_2_ gene expression ratios on day 2 or day 4 vs. day 0.
(XLSX)

Table S4 **Detailed information on DEGs within four significant expression profiles during MSVC differentiation.** Four gene expression profiles (profiles 11, 14, 4, and 1) were significantly enriched in MSVC differentiation. DEGs in each gene expression profile were shown. The gene expression levels were normalized by the log_2_ gene expression ratios on day 2 or day 4 vs. day 0.
(XLSX)

Table S5 **Q-PCR primers of mRNAs.**
(DOCX)

Table S6 **Relative expression levels of the selected eight genes by Q-PCR.**
(DOCX)
