# Supplementary figures and images for: Transcriptome Comparison between Porcine Subcutaneous and Intramuscular Stromal Vascular Cells during Adipogenic Differentiation
Source: PLoS One. 2013 Oct 10;8(10):e77094. doi: 10.1371/journal.pone.0077094 (PMC3795010; doi:10.1371/journal.pone.0077094)

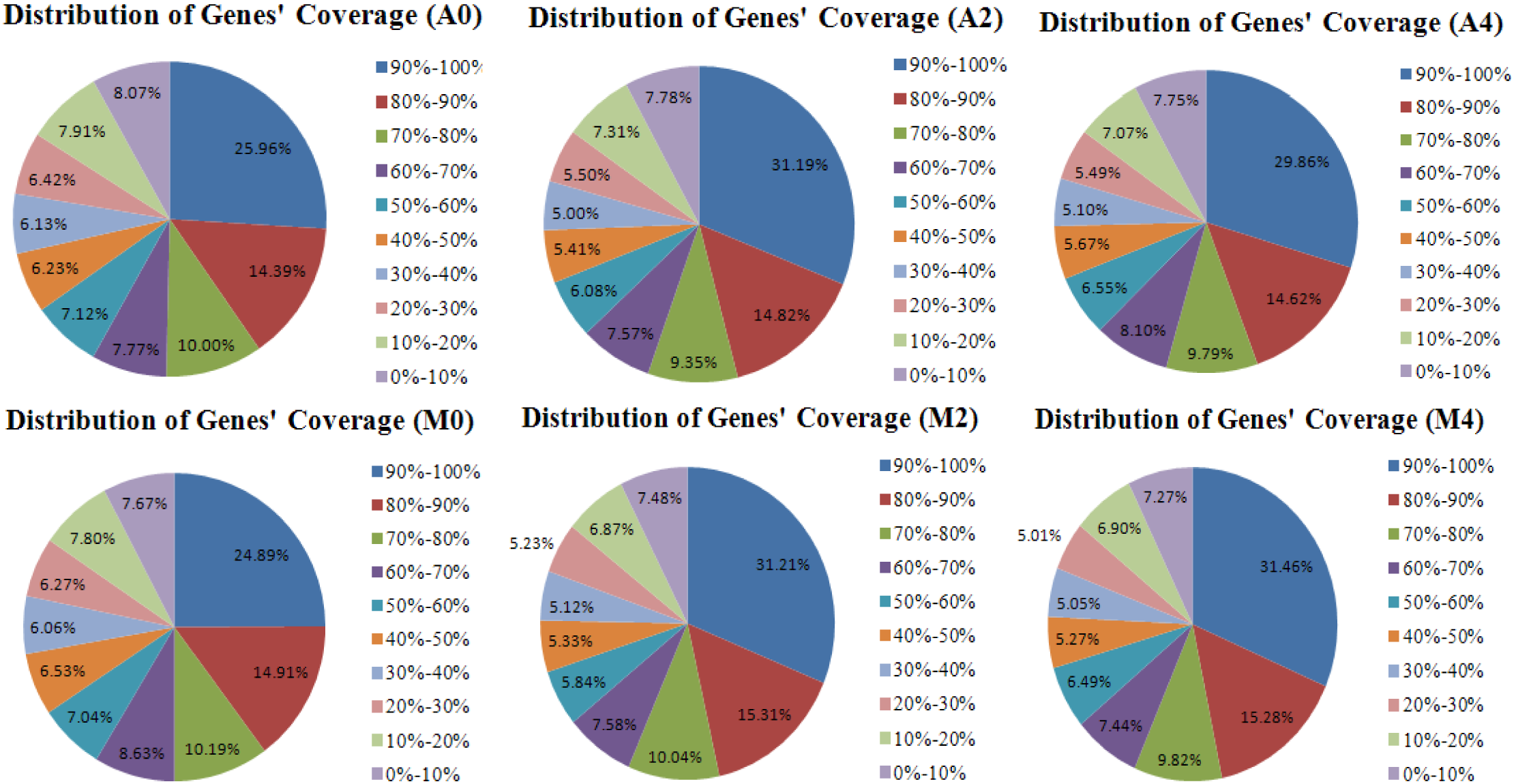

Supplement: Figure S1 — Percentage coverage representing the percentage of genes expressed in each of the six samples mapping in the pig genome. A0, ASVC differentiation on day 0; A2, ASVC differentiation on day 2; A4, ASVC differentiation on day 4; M0, MSVC differentiation on day 0; M2, MSVC differentiation on day 2; M4, MSVC differentiation on day 4. Gene coverage is the percentage of a gene covered by reads. This value is equal to the ratio of the base number in a gene covered by mapping reads to the total bases number of that gene. The distribution of distinct reads over different read abundance categories show similar patterns for all six RNA-Seq libraries. (TIF) [file pone.0077094.s001.tif]
