# Supplementary material for: Transcriptome Comparison between Porcine Subcutaneous and Intramuscular Stromal Vascular Cells during Adipogenic Differentiation
Source: PLoS One. 2013 Oct 10;8(10):e77094. doi: 10.1371/journal.pone.0077094 (PMC3795010; doi:10.1371/journal.pone.0077094)
Supplement: Table S1 — Size distribution of gene sequences detected in ASVC and MSVC differentiation using RNA-Seq. (DOCX) [file pone.0077094.s002.docx]

**Table S1. Size distribution of gene sequences detected in ASVC and MSVC differentiation using RNA-Seq.**

| **Gene length (bp)** | **Total number of genes** | **Percentage (%)** |
| --- | --- | --- |
| **100-500** | **724** | **3.56** |
| **500-1000** | **3632** | **17.84** |
| **1000-1500** | **4029** | **19.80** |
| **1500-2000** | **3635** | **17.86** |
| **≥2000** | **8333** | **40.94** |
| **Total** | **20353** | **100** |
