# Supplementary material for: Transcriptome Comparison between Porcine Subcutaneous and Intramuscular Stromal Vascular Cells during Adipogenic Differentiation
Source: PLoS One. 2013 Oct 10;8(10):e77094. doi: 10.1371/journal.pone.0077094 (PMC3795010; doi:10.1371/journal.pone.0077094)
Supplement: Table S5 — Q-PCR primers of mRNAs. (DOCX) [file pone.0077094.s006.docx]

| Gene Symbol | Accession number | Sense primer (5’-3’) | Anti-sense primer (5’-3’) | Product |
| --- | --- | --- | --- | --- |
| S100A8 | NM_001160271.3 | GCGGATGACTTGAAGAGATTGTTAG | GCTCTGCTACTCTTTGTGGATGTCT | 186 |
| S100A12 | NM_001160272.1 | GGCATTATGACACCCTTATC | GTCACCAGGACCACGAAT | 169 |
| KLF15 | NM_001134349.2 | GCATGGTGGACCACTTGCTT | CAAAGGGCTTGCGAGTCAGG | 184 |
| KLF13 | NM_001011505.1 | CGGGCTGTGAGAAAGTTTACGG | ATGAAGCGTTTGTCGCAGATGG | 196 |
| EGR1 | XM_003123974.3 | CTCTTAGGTGGGATGGAGGTT | AGCTGAAGTCAAAGGGAGTAGG | 142 |
| TSC22D3 | XM_003135298.3 | CGAAATGTATCAGACCCCCA | GGCTCCAGAGGCACTGTTAT | 127 |
| C/EBPβ | NM_001199889.1 | GTCCAAACCAACCGCACAT | GAAACAACCCCGTAGGAACAT | 262 |
| ZBTB16 | XM_003357303.1 | GGGGACAAGGTTGAGGAAAGG | CGGAGTAGATGCCCAGGTGC | 202 |
| β-actin | XM_003124280.2 | CCAGGTCATCACCATCGG | CCGTGTTGGCGTAGAGGT | 158 |
