# Supplementary material for: Transcriptome Comparison between Porcine Subcutaneous and Intramuscular Stromal Vascular Cells during Adipogenic Differentiation
Source: PLoS One. 2013 Oct 10;8(10):e77094. doi: 10.1371/journal.pone.0077094 (PMC3795010; doi:10.1371/journal.pone.0077094)
Supplement: Table S6 — Relative expression levels of the selected eight genes by Q-PCR. (DOCX) [file pone.0077094.s007.docx]

**Table S6. Q-PCR validation of RNA-Seq data on a selected of eight genes**

| Transcript | Gene symbol | Cell type | ASVC and MSVC adipogenic differentiation | | | *P* value |
| --- | --- | --- | --- | --- | --- | --- |
|  |  |  | Day 0 | Day 2 | Day 4 |  |
| NM_001160271.3 | S100A8 | ASVC | 1.09±0.13^c^ | 13.66±2.28^a^ | 5.67±0.79^b^ | < 0.01 |
|  |  | MSVC | 1.31±0.30^c^ | 80.36±4.92^a^ | 18.00±2.38^b^ | < 0.01 |
| NM_001160272.1 | S100A12 | ASVC | 1.10±0.10^c^ | 8.48±0.24^a^ | 5.49±0.42^b^ | < 0.01 |
|  |  | MSVC | 1.97±0.75^c^ | 44.36±5.59^a^ | 14.40±2.68^b^ | < 0.01 |
| NM_001134349.2 | KLF15 | ASVC | 1.13±0.11^c^ | 12.06±0.97^a^ | 6.76±0.60^b^ | < 0.01 |
|  |  | MSVC | 1.11±0.04^c^ | 20.52±0.63^a^ | 13.51±0.34^b^ | < 0.01 |
| NM_001011505.1 | KLF13 | ASVC | 6.22±1.02^b^ | 5.92±0.67^b^ | 9.42±0.84^a^ | < 0.01 |
|  |  | MSVC | 5.35±0.48^b^ | 21.79±1.69^a^ | 21.31±0.98^a^ | < 0.01 |
| XM_003123974.3 | EGR1 | ASVC | 1.48±0.20^b^ | 3.86±0.29^a^ | 1.27±0.27^b^ | < 0.01 |
|  |  | MSVC | 5.31±0.26^a^ | 2.63±0.28^b^ | 1.58±0.14^c^ | < 0.01 |
| XM_003135298.3 | TSC22D3 | ASVC | 1.10±0.06^c^ | 11.25±0.91^a^ | 6.31±0.56^b^ | < 0.01 |
|  |  | MSVC | 1.38±0.47^c^ | 19.14±0.59^a^ | 12.44±0.60^b^ | < 0.01 |
| NM_001199889.1 | C/EBPβ | ASVC | 1.26±0.23^b^ | 1.42±0.39^b^ | 3.11±0.78^a^ | < 0.01 |
|  |  | MSVC | 3.59±0.01^b^ | 7.72±1.35^a^ | 3.42±0.70^b^ | < 0.01 |
| XM_003357303.1 | ZBTB16 | ASVC | 2.76±1.17^b^ | 54.52±4.16^a^ | 4.87±0.56^b^ | < 0.01 |
|  |  | MSVC | 5.42±1.79^b^ | 14.56±2.23^a^ | 4.85±0.09^b^ | < 0.01 |

The analytical data were presented in the table as ‘Means ± S.D’. Means in the same row without common superscripts differ. Difference between means was considered significant if *P* < 0.05, and extremely significant if *P* < 0.01.
